# Evaluation of different antibiotic prophylaxis strategies for hepatectomy: A network meta-analysis

**DOI:** 10.1097/MD.0000000000016241

**Published:** 2019-06-28

**Authors:** Tao Guo, Ruiwen Ding, Jian Yang, Ping Wu, Pengpeng Liu, Zhisu Liu, Zhen Li

**Affiliations:** a Department of Hepatobiliary and Pancreatic Surgery; b Department of Anesthesiology, Zhongnan Hospital of Wuhan University, Wuhan; c School of Nursing, Huanggang Polytechnic College, Huanggang, PR China.

**Keywords:** antibiotic prophylaxis, hepatectomy, network meta-analysis

## Abstract

**Background:**

The application of antibiotic prophylaxis for hepatectomy remains uncertain. This research aims to evaluate different antibiotic prophylaxis strategies for hepatectomy based on network meta-analysis.

**Methods:**

Literature retrieval was conducted in globally recognized databases, namely, MEDLINE, EMBASE and Cochrane Central, to address relative randomized controlled trials (RCTs) investigating antibiotic prophylaxis strategies for hepatectomy. Relative parametric data, including surgical site infection (SSI), remote site infection (RSI) and total infection (TI), were quantitatively pooled and estimated based on the Bayesian theorem. The values of surface under the cumulative ranking curve (SUCRA) probabilities regarding each parameter were calculated and ranked. Node-splitting analysis was performed to test the inconsistency of the main results, and publication bias was assessed by examining the funnel plot symmetry. Additional pairwise meta-analyses were performed to validate the differences between respective strategies at the statistical level.

**Results:**

After a detailed review, a total of 5 RCTs containing 4 different strategies were included for the network meta-analysis. The results indicated that the application of no antibiotics possessed the highest possibility of having the best clinical effects on SSI (SUCRA, 0.56), RSI (SUCRA, 0.46) and TI (SUCRA, 0.61). Moreover, node-splitting analysis and funnel plot symmetries illustrated no inconsistencies in the current study. Additional pairwise meta-analyses determined that additional and long-duration applications had no clinical benefit.

**Conclusion:**

Based on current evidence, we concluded that antibiotic prophylaxis did not reveal clinical benefit in hepatectomy. However, more relative trials and statistical evidence are still needed.

## Abstract 2

Supplemental Digital Content is available in the text

## 1 Introduction

Hepatectomy is an established treatment modality for benign and malignant diseases of the liver. With recent improvements in perioperative management and surgical techniques, hepatectomy has become a safe surgical procedure that has only 0% to 4% mortality rates.^[1–3]^ Nevertheless, postoperative infections must be considered because postoperative impaired hepatic function contributes to a high risk of surgical site infection (SSI).^[4,5]^ Moreover, hepatectomy is classified into a clean-contaminated surgery because the bile duct is dissected, and this exposure renders the patient more susceptible to infection. Therefore, the application of antibiotic prophylaxis has been clinically considered for the last 2 decades. On the other hand, antibiotic prophylaxis has been demonstrated to be unnecessary in clean surgeries but seemed to be effective among gastroenterologic operations, which are also regarded as a clean-contaminated surgery.^[6,7]^ Interestingly, several studies also discovered that postoperative antibiotic prophylaxis is unnecessary for laparoscopic cholecystectomy, which is another clean-contaminated surgery.^[8,9]^ Therefore, some unreliable factors exist in clinical references for the guidance of antibiotic prophylaxis for hepatectomy.

More importantly, in the last 2 decades, several randomized controlled trials (RCTs) investigating the efficacy of antibiotic prophylaxis on hepatectomy were published from different regions with contradictory results. Furthermore, although these trials tried to discover the clinical efficacy of antibiotic prophylaxis, they were conducted with multifarious strategies (such as preoperative application and postoperative long- or short-term duration). Until now, no consensus on the superior prophylactic antibiotic strategy for hepatectomy and no quantitative network estimation have been conducted to comprehensively evaluate these antibiotic prophylaxis strategies. Therefore, the current network meta-analysis aimed to determine pooled estimations of antibiotic prophylaxis strategies and undertook the purpose of providing objective evidence for clinical decision-making.

## 2 Methods

### 2.1 Literature search and retrieval

Current meta-analysis was based entirely on previous published studies which had declared ethical approvals and no original clinical raw data was collected or utilized, thereby ethical approval was not conducted for this study. Our review was performed according to previously established Preferred Reporting Items for Systematic Reviews and Meta-Analyses (PRISMA) guidelines^[10]^ and was pre-registered in PROSPERO (CRD42019121084). Literature retrieval was conducted in a globally recognized electronic database, namely, MEDLINE, EMBASE, and Cochrane Central, to achieve the optimal raw data. Relative mesh items and their combinations were applied to address relevant trials investigating antibiotic prophylaxis for hepatectomy (example retrieval strategy in MEDLINE is presented in Supplementary Table S1). One requirement was that the full text had to be in English; however, the publication status and date were not restricted.

### 2.2 Inclusion and exclusion criteria

Two researchers independently reviewed the title and abstract of each assay to select studies for further screening if meeting the following criteria:

(1) RCTs;
(2) trials focused on antibiotic prophylaxis strategies for hepatectomy;
(3) antibiotic prophylaxis strategy was the only intervention;
(4) studies providing at least 1 available parameter of interest.

Meanwhile, the following items were defined as exclusion criteria:

(1) not an RCTs;
(2) no available parametric data reported;
(3) reviews, comments, case reports or study protocols;
(4) studies focusing on basic science;
(5) trials with insufficient raw data;
(6) vague strategy or mixed diseases.

### 2.3 Raw data extraction and quality assessment

General information (e.g., author name, publication data and region) and intervention-related characteristics (e.g., sample size and reported parameters) were extracted using a predesigned form. In the current study, we aimed to investigate the efficacy of various antibiotic prophylaxis strategies for hepatectomy; thus, relevant parametric data of infections were selected for pooled estimation. We included SSI,^[11]^ remote site infection (RSI)^[12]^ and total infection (TI) rate (including any signs and symptoms related to infection, such as systemic inflammatory response syndrome, bacteremia, etc, in addition to SSI and RSI) as parametric data to make a comprehensive judgment for the evaluation of the feasibility and efficacy of different antibiotic prophylaxis strategies.

In addition, the included trials were assessed by the Cochrane Risk of Bias assessment tool^[13]^ to clarify the relative bias risk of individual studies with the following requirements:

(1) free of selection bias;
(2) free of performance bias;
(3) free of detection bias;
(4) free of attrition bias;
(5) free of reporting bias; and
(6) free of other biases.

Relative graphics of bias risks for all included trials and the judgment for each trial were rated by Review Manager software (version 5.3).

The raw data extraction and bias risk assessments were independently conducted by 2 investigators. Any disagreements were resolved by a group discussion with all team members.

### 2.4 Statistical analysis

We aimed to evaluate different antibiotic prophylaxis strategies for hepatectomy in the current study; therefore, a quantitative network comparison based on the Bayesian theorem was necessary. This statistical procedure incorporates both direct and indirect information through a common comparator to obtain estimates of the relative interventional effects on multiple intervention comparisons.^[14,15]^ The values of surface under the cumulative ranking curve (SUCRA) probabilities based on the consistency model are presented to clarify the pros and cons of different strategies. The highest SUCRA values represented the probability of achieving the best clinical effects regarding each parameter.^[16,17]^ Odds ratios (ORs) and related 95% credible intervals (CIs) derived from network meta-analysis were calculated to compare different strategies. Both consistency and inconsistency model approaches were used to detect the reliability of the main results. Node-splitting analysis was conducted for closed loop calculation, and no statistical inconsistencies were shown if *P* > .05.^[18]^ Publication bias was assessed by examining the funnel plot symmetry. However, a pairwise meta-analysis was also conducted if additional evidence was needed. In this condition, heterogeneity (*I*^2^, index statistic) in the study design was used to estimate a data mode by using fixed (*I*^2^ < 50%) or random (*I*^2^ > 50%) effects models.^[19]^ The associated 95% CIs were calculated, and the level of statistical significance was set at *P* < .05. Data manipulation, statistical analyses of network meta-analysis and pairwise analyses were conducted using Aggregate Data Drug Information System automated software (ADDIS, version 1.16) and the Stata software package (version 12.0).

## 3 Results

### 3.1 Study characteristics and quality assessment

**Figure 1:**
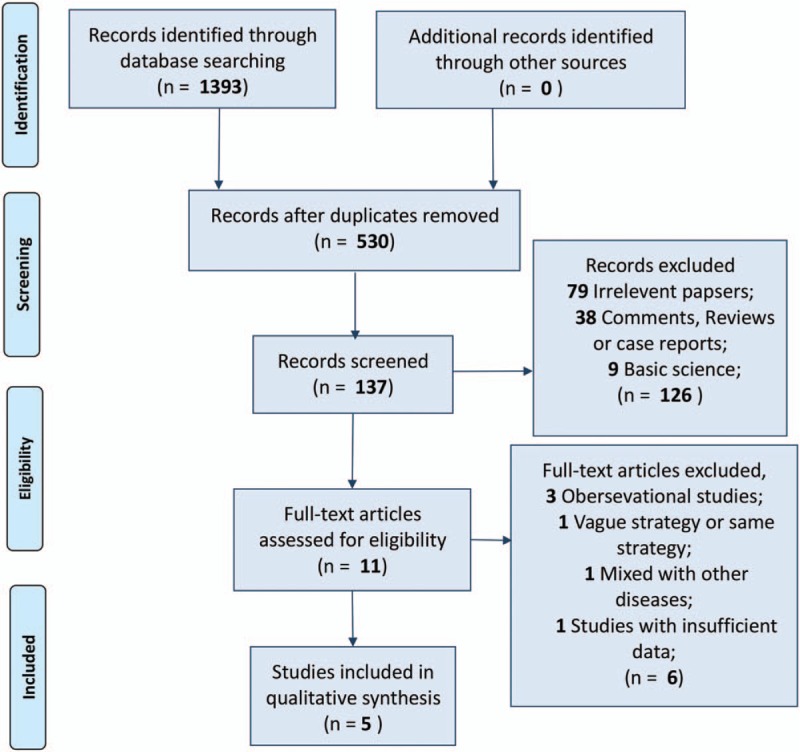
Flow diagram of the process of selecting studies for the current network meta-analysis.

**Figure 2:**
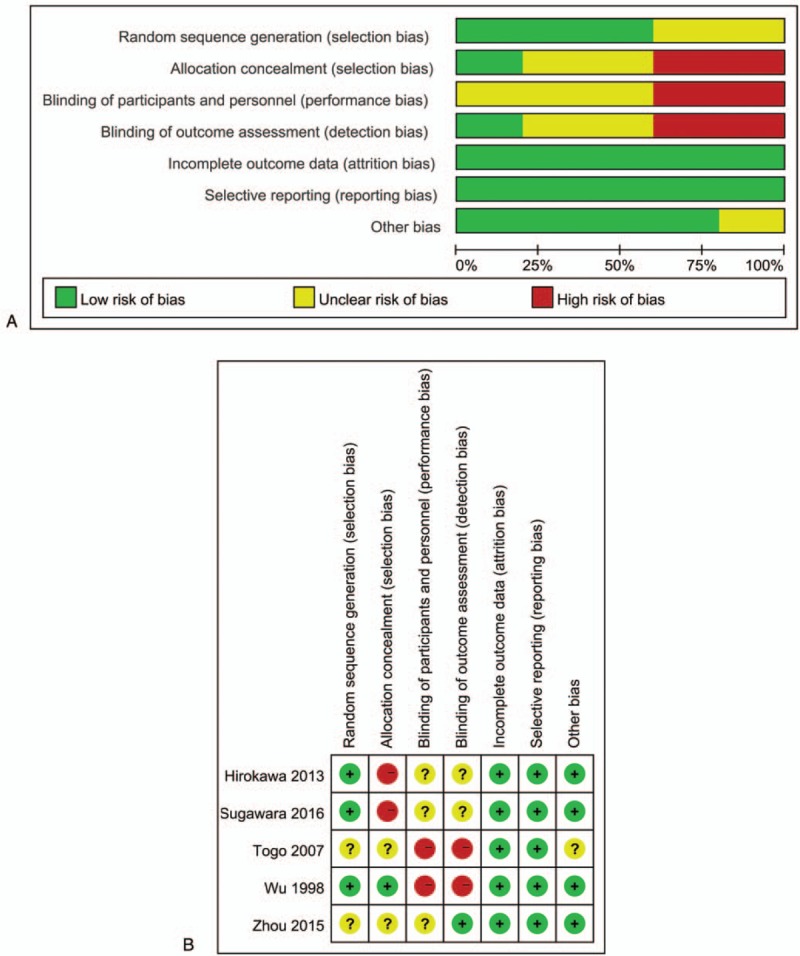
Bias assessments for included trials. (A) Risk of biases graph presented as percentages across all of the included studies; (B) Judgments regarding each risk of bias item for each included study.

After carefully review, 5 RCTs containing 701 patients were included for the final evaluation^[20–24]^ (Fig. 1). These 5 papers were all published from Asia, and each of them provided sufficient raw parametric data of interest. Among the included 5 trials, 4 antibiotic prophylaxis strategies were introduced, namely, preoperative application (PRA, defined as short-duration administration of antibiotics prior to skin incision), postoperative short-duration application (POS, defined as ≤2 days postoperative application), postoperative long-duration application (POL, defined as >2 days postoperative application), negative control (NC, indicating no antibiotic prophylaxis) and their combinations (Table 1). At the same time, 3 of the included trials reported clear random consequence generation, yet allocation concealment and a clear blind procedure were barely mentioned (Fig. 2).

**Table 1:**
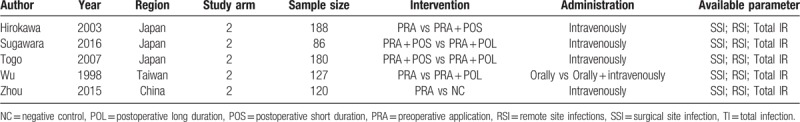
Characteristics of included studies.

### 3.2 Results of the network meta-analysis

**Figure 3:**
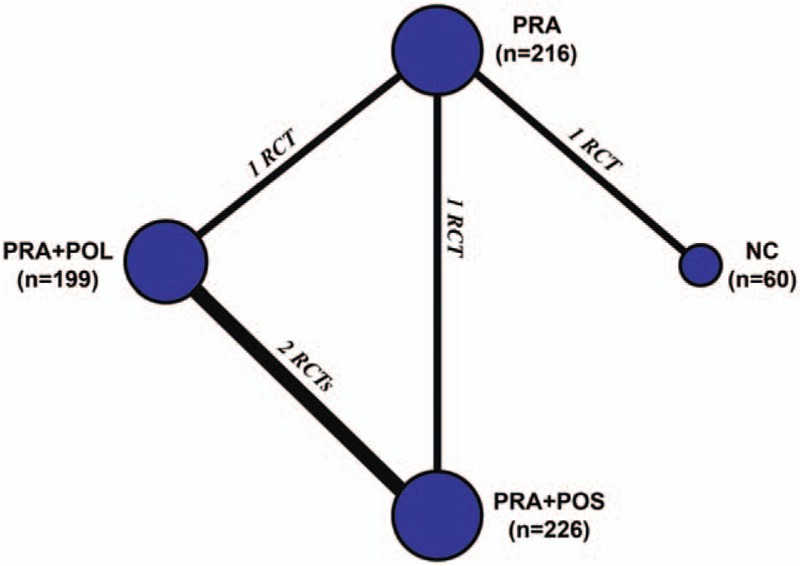
Network connections of all of the included trials. The numbers on the line indicate the quality of studies compared with every pair of strategies, which are also represented by the width of the lines. Additionally, the sizes of the areas of the circles indicate the respective sample sizes. PRA = preoperative application, POL = postoperative long-duration, POS = postoperative short-duration, NC = negative control.

**Figure 4:**
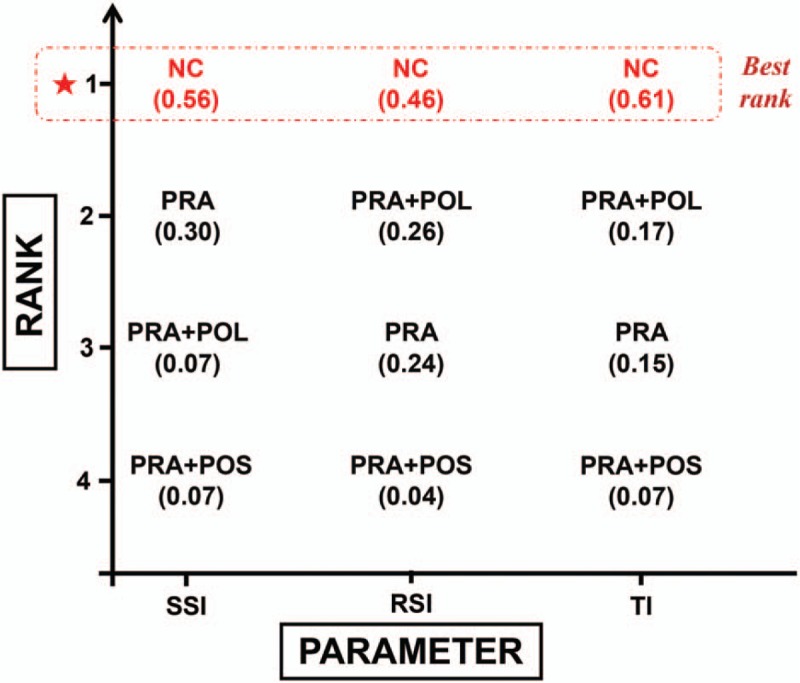
Plot of surface under the cumulative ranking curve (SUCRA) values of respective strategies regarding different parameters.

We performed quantitative pooled estimations based on network connections of included trials regarding SSI, RSI, and TI (Fig. 3). All included 5 trials reported the 3 outcomes of interest. After quantitative pooled estimates, the results of the network meta-analysis revealed that the application of no antibiotic prophylaxis (NC) exhibited the highest probability of achieving the lowest rate of SSI (SUCRA, 0.56), RSI (SUCRA, 0.46), and TI (SUCRA, 0.61) (Fig. 4) (Supplementary Table S2). Therefore, we concluded that no antibiotic application was potentially the best antibiotic prophylaxis strategy to prevent postoperative infections.

### 3.3 Data consistency and publication bias

According to previous results, we demonstrated that no antibiotic prophylaxis seemed to be the superior strategy to reduce postoperative infection rates. To test the reliability of the main results, we made comparisons based on the inconsistency model, and the results were similar compared with the consistency model (Supplementary Table S3). Moreover, the results of the node-splitting model calculation illustrated that no data inconsistency existed in our results with all calculations have a *P* > .05 (Supplementary Table S4). Moreover, funnel plot symmetries regarding SSI (Supplementary Fig. S1), RSI (Supplementary Fig. S2) and TI (Supplementary Fig. S3) did not detect obvious bias in current study.

### 3.4 Additional analysis

**Figure 5:**
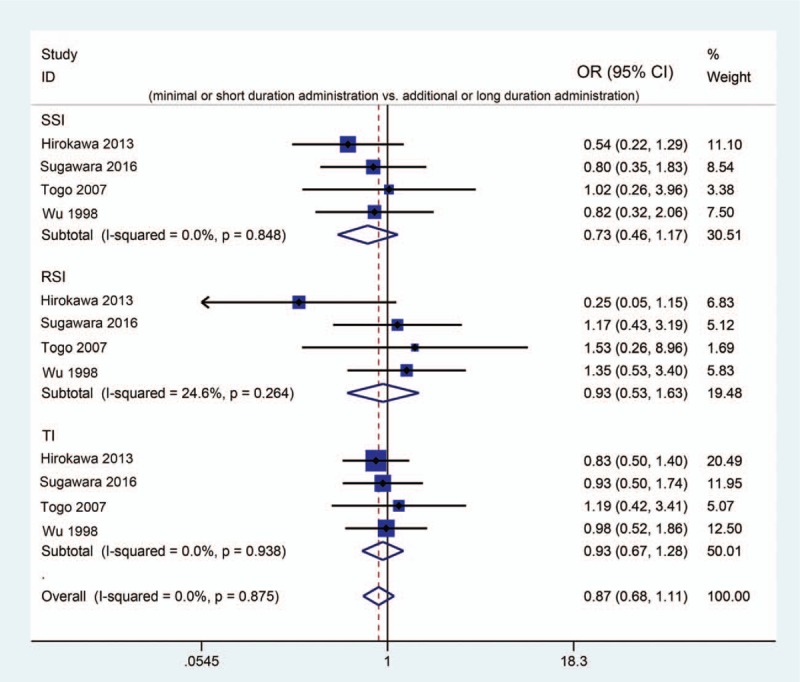
Forest plot of pairwise meta-analysis comparing non-additional or short-duration administration and additional or long-duration administrations.

Unexpectedly, the results of the network meta-analysis determined that the strategy of no antibiotic prophylaxis application revealed the highest probability of achieving the best clinical effects for hepatectomy regarding all selected parametric data. To further verify this result at the statistical level, we conducted pairwise comparisons between NC and non-NC strategies. However, for all included 5 trials, only 1 trial reported NC-related raw data, which revealed no significant difference compared with PRA.^[24]^ This implied that although NC may possess the highest probability of achieving the best clinical for lowering postoperative infection, there was no direct statistical evidence to support this. In addition, if we determined that NC is the potential superior way, whether additional or long-duration antibiotic administration brings adverse clinical efficacy should be determined. Thus, we used another pairwise meta-analysis to clarify whether additional antibiotics causes adverse effects compared to less- or short-duration antibiotic administration. Four included trials exhibited relative comparisons.^[20–23]^ Based on a fixed model (*I*^2^ < 50% for all), we discovered no significant differences between non-additional or less antibiotic administration and additional or long-duration administration regarding postoperative SSI (OR [95% CI] = 0.73 [0.46, 1.17]; Test *Z* = 1.29; *P* = .196), RSI (OR [95% CI] = 0.93 [0.53, 1.63]; Test *Z* = 0.25; *P* = .803), and TI (OR [95% CI] = 0.93 [0.67, 1.28]; Test *Z* = 0.45; *P* = .654) (Fig. 5). Currently, we concluded that additional or more antibiotic administration revealed no clinical benefits for hepatectomy and is not recommended. Therefore, antibiotic prophylaxis did not provide superior clinical efficacy for preventing postoperative infection; however, more statistical evidence is needed.

## 4 Discussion

The current network meta-analysis aimed to evaluate different antibiotic prophylaxis strategies by quantitatively comparing SSI, RSI, and TI based on the Bayesian theorem. In total, 5 RCTs containing 701 patients were included. Among these trials, 4 different antibiotic prophylaxis strategies were reported, and 3 infection-related parameters were selected for pooled estimation. Surprisingly, the main results revealed that application of no antibiotic prophylaxis exhibited the highest probability of achieving the best clinical effects regarding all included parameters. This indicated that antibiotic prophylaxis may bring adverse efficacy; thus, we performed a pairwise meta-analysis to determine whether additional or more antibiotic administration causes adverse effects at the statistical level. The results revealed no significant differences between antibiotic prophylaxis strategies regarding all infection-related parameters.

We know that hepatectomy is considered a major operation due to its long operation time and massive blood loss, although it is not a heavily contaminated procedure.^[25]^ Large dead spaces and areas of devitalized tissue on the cut surface are present. If contaminated with bile or bacteria, intraabdominal infection readily occurs.^[26]^ Meanwhile, intestinal bacteria translocation, postoperative inserted catheters and urinary output monitoring are also high-risk factors for infections. Therefore, different antibiotic prophylaxis strategies have been widely applied for hepatectomy, yet no comprehensive evaluation has been reported. A previous Cochrane pairwise meta-analysis tried to assess different methods to decrease infection after liver resections.^[27]^ This paper included antibiotics, prebiotics, probiotics, recombinant bactericidal-permeability increasing protein (RBPIP) and topical betadine gel as experimental arms versus placebo as the control arm. The authors of this meta-analysis claimed no evidence to support or refute the use of any treatment to reduce infectious complications after liver resections. This paper made an innovative attempt to assess different methods for reducing post-hepatectomy infections. However, this study failed to perform quantitative network estimation, and the classification of methods, especially for antibiotic prophylaxis strategies, lacked accurateness and meticulousness, which may not reveal the essential roles of different strategies. More importantly, only 2 antibiotic-related trials were included at that time, and the authors did not raise explicit future research directions. Therefore, it was necessary to perform a comprehensive network evaluation for different antibiotic prophylaxis strategies with accurate descriptions. Unlike those simple pairwise meta-regressions, our study comprehensively assessed all reported antibiotic prophylaxis strategies with accurate descriptions and classifications. We discovered that the application of no antibiotic prophylaxis possessed the highest probability of achieving the lowest postoperative infection rate based on the Bayesian theorem. This result was an unexpected discovery. This finding indicated that the application of antibiotic prophylaxis may potentially increase the risk of postoperative infection. Nevertheless, this speculation could not be easily determined due to inadequate statistical evidence while it may enlighten us in clinical reviews. Previous trials have demonstrated that antibiotic prophylaxis has limited clinical benefits for clean or clean-contaminated surgeries.^[8,9,28–30]^ These findings may imply that progressively improved surgical techniques and other non-antibiotic-based physical prophylactic procedures are more important because infection complications are usually related to technical pitfalls rather than the use of prophylactic antibiotics. With the development of aseptic and minimally invasive procedures, antibiotic-dependent perioperative management has become increasingly less important. Moreover, antibiotic administration may lead to dysbacteria and double infections. Our results also demonstrated that additional or long-duration antibiotics revealed no clinical benefits. Thus, hepatectomy without antibiotic prophylaxis may be a potential superior method for future perioperative management. On the other hand, as we mentioned above, application of no antibiotic prophylaxis revealed potential best clinical effects, but it contains inadequate statistical support. Only 1 RCT illustrated that no antibiotic prophylaxis revealed a similar benefit compared with preoperative antibiotic administration.^[24]^ Therefore, although the Bayesian theorem confirms the superiority of no antibiotic prophylaxis, its essential role was not accurately addressed and it probably exhibited no difference with other strategies. Nevertheless, this study contained sample size of 701 patients which were separated into 4 arms, thereby, to some extent, the results exhibited some guiding significance. Notably, current meta-analysis aimed to finish the first attempt to provide relative clinical evidence about antibiotic prophylaxis strategies. We now addressed that application of no antibiotic prophylaxis may be potentially the superior strategy although more data was needed. Therefore, at mean time, we also raised relative clinical research direction whether antibiotic prophylaxis should be used in hepatectomy. What is more, we summarize that long-duration and additional application of antibiotics revealed no benefit and are not recommended. Thus, investigations comparing no antibiotic administration and less- or short-duration antibiotic applications should be performed in the future. Whether preoperative or postoperative short-duration antibiotic administration should be applied and even whether perioperative antibiotics are still valuable are urgently needed. In summary, these topics should be the directions of future clinical research.

To the best of our knowledge, the current study is the first network meta-analysis evaluating various antibiotic prophylaxis strategies for hepatectomy. We made comprehensive estimations and raised directions for future research. Nevertheless, we must admit some limitations exist in our study. First, 4 different strategies were analyzed from 701 patients. However, only 5 trials were included, and some crucial pairwise analyses could not be performed; therefore, we expect more trials in the future. Second, although the node-splitting model calculation demonstrated that no data inconsistencies existed in our main results and funnel plots did not detected obvious bias, all included trials were reported from Asia and we noticed some high risk items existed in current study (Fig. 2); therefore, we believe that some potential local bias may exist in our study. Additionally, no clear double-blind procedures were reported in the included trials, which may bring undetected confounding factors. Finally, we only focused on the infection rate as parametric data without comparing other parameters, such as antibiotic-related adverse events, due to insufficient raw data. This is another reason why more trials are urgently needed.

In summary, the current meta-analysis demonstrated that the application of no prophylactic antibiotics may be the potential superior strategy for decreasing the postoperative infections. However, these results need more trials for further validation at the statistical level. Moreover, additional and long-duration administration of antibiotics exhibited no clinical benefit for hepatectomy and are not recommended. More importantly, the different effects between short-duration or less application and no application should be addressed in the future. Therefore, at present, antibiotic prophylaxis did not revealed clinical benefit in hepatectomy and more trails in this direction were expected in the future.

## Author contributions

**Conceptualization:** Tao Guo, Zhen Li.

**Data curation:** Tao Guo, Ruiwen Ding, Jian Yang, Pengpeng Liu.

**Formal analysis:** Ping Wu.

**Funding acquisition:** Ping Wu.

**Investigation:** Tao Guo, Jian Yang, Ping Wu.

**Methodology:** Tao Guo, Jian Yang, Ping Wu.

**Project administration:** Zhisu Liu, Zhen Li.

**Resources:** Tao Guo, Ping Wu.

**Software:** Tao Guo, Jian Yang, Ping Wu, Pengpeng Liu.

**Supervision:** Zhisu Liu.

**Validation:** Tao Guo, Ruiwen Ding, Jian Yang, Pengpeng Liu, Zhisu Liu.

**Visualization:** Tao Guo, Zhisu Liu.

**Writing – original draft:** Tao Guo, Zhen Li.

**Writing – review & editing:** Tao Guo, Ruiwen Ding, Zhen Li.

## Supplementary Material

Supplemental Digital Content
