# Supplementary material for: Evaluation of different antibiotic prophylaxis strategies for hepatectomy: A network meta-analysis
Source: Medicine (Baltimore). 2019 Jun 28;98(26):e16241. doi: 10.1097/MD.0000000000016241 (PMC6617204; doi:10.1097/MD.0000000000016241)

Supplementary Tables and Figures

Table S1. Example search strategy and process in MEDLINE.

# No.	Search	Results
1	exp (hepatic) OR liver/	1128322
2	exp (resection) OR excision/	380390
3	exp hepatectomy	34427
4	exp (surgery) OR operation/	4493358
5	1 AND 2	39182
6	1 AND 4	260248
7	(5 OR 6) AND 3	30689
8	exp (prospective) OR random/	1181896
9	exp randomized controlled trial/	609695
10	8 OR 9	1646763
11	exp (prophylactic antibiotic) OR antibiotic prophylaxis/	27206
12	10 AND 11	6106
13	7 AND 12	64

Table S2. Comparisons of included strategies in network meta-analysis regarding infection rates based on consistency model. Odds ratios are presented in the cells in common between the column-defining and row-defining strategies. [OR: odds ratio; CI: credible intervals].

**Parameter**	**Comparison [OR (95%CI)]**			
**SSI**	NC	1.15 (0.31, 3.84)	1.93 (0.37, 8.70)	1.87 (0.38, 8.43)
	0.87 (0.26, 3.26)	PRA	1.65 (0.59, 4.80)	1.64 (0.61, 4.23)
	0.52 (0.12, 2.72)	0.60 (0.21, 1.68)	PRA+POL	0.97 (0.41, 2.29)
	0.53 (0.12, 2.62)	0.61 (0.24, 1.65)	1.03 (0.44, 2.45)	PRA+POS
**RSI**	NC	1.13 (0.19, 7.51)	1.31 (0.14, 15.40)	2.52 (0.25, 30.17)
	0.89 (0.13, 5.18)	PRA	1.14 (0.27, 5.27)	2.23 (0.51, 11.06)
	0.76 (0.06, 7.13)	0.88 (0.19, 3.74)	PRA+POL	1.93 (0.53, 7.28)
	0.40 (0.03, 3.94)	0.45 (0.09, 1.95)	0.52 (0.14, 1.88)	PRA+POS
**TI**	NC	1.28 (0.51, 3.03)	1.40 (0.47, 4.25)	1.50 (0.53, 4.31)
	0.78 (0.33, 1.94)	PRA	1.10 (0.61, 2.17)	1.17 (0.68, 2.19)
	0.71 (0.24, 2.13)	0.91 (0.46, 1.65)	PRA+POL	1.08 (0.56, 1.98)
	0.67 (0.23, 1.90)	0.85 (0.46, 1.46)	0.93 (0.51, 1.78)	PRA+POS

Table S3. Network meta-analysis comparing different antibiotic prophylaxis strategies based on inconsistency model. [OR: odds ratio; CI: credible intervals].

**Parameter**	**Comparison [OR (95%CI)]**			
**SSI**	NC	1.11 (0.35, 3.92)	1.82 (0.39, 9.36)	1.73 (0.37, 9.71)
	0.90 (0.26, 2.86)	PRA	1.58 (0.61, 4.55)	1.63 (0.62, 4.46)
	0.55 (0.11, 2.54)	0.63 (0.22, 1.64)	PRA+POL	0.95 (0.40, 2.26)
	0.58 (0.10, 2.69)	0.61 (0.22, 1.61)	1.05 (0.44, 2.50)	PRA+POS
**RSI**	NC	1.18 (0.20, 6.95)	1.20 (0.13, 13.04)	2.12 (0.21, 26.90)
	0.84 (0.14, 4.95)	PRA	1.05 (0.25, 4.99)	2.58 (0.55, 13.62)
	0.84 (0.08, 7.48)	0.96 (0.20, 3.95)	PRA+POL	1.80 (0.51, 6.70)
	0.47 (0.04, 4.66)	0.39 (0.07, 1.81)	0.56 (0.15, 1.98)	PRA+POS
**TI**	NC	1.27 (0.50, 3.11)	1.41 (0.45, 4.23)	1.51 (0.50, 4.32)
	0.79 (0.32, 1.99)	PRA	1.10 (0.59, 2.18)	1.21 (0.66, 2.20)
	0.71 (0.24, 2.21)	0.91 (0.46, 1.68)	PRA+POL	1.07 (0.58, 1.95)
	0.66 (0.23, 2.02)	0.83 (0.45, 1.53)	0.93 (0.51, 1.73)	PRA+POS

Table S4. Results of node-splitting models by testing the direct and indirect effects. Potential inconsistency may exist if P < 0.05.

Parameter	Item	Direct Effect	Indirect Effect	Overall	P-Value
SSI	PRA vs. PRA+POL	0.21 (-1.10, 1.59)	0.89 (-0.67, 2.46)	0.50 (-0.52, 1.57)	0.53
	PRA vs. PRA+POS	0.67 (-0.48, 1.92)	0.10 (-1.62, 1.68)	0.50 (-0.50, 1.44)	0.61
	PRA+POL vs. PRA+POS	-0.18 (-1.20, 0.83)	0.52 (-1.26, 2.34)	-0.03 (-0.89, 0.83)	0.50
RSI	PRA vs. PRA+POL	-0.32 (-2.15, 1.39)	1.35 (-1.31, 4.18)	0.13 (-1.32, 1.66)	0.27
	PRA vs. PRA+POS	1.60 (-0.43, 4.14)	-0.08 (-2.42, 2.19)	0.80 (-0.67, 2.40)	0.24
	PRA+POL vs. PRA+POS	0.41 (-1.09, 1.65)	1.70 (-0.53, 4.69)	0.66 (-0.63, 1.98)	0.19
TI	PRA vs. PRA+POL	0.02 (-0.80, 0.84)	0.18 (-0.93, 1.23)	0.09 (-0.50, 0.78)	0.80
	PRA vs. PRA+POS	0.24 (-0.45, 0.96)	0.01 (-1.12, 1.19)	0.16 (-0.38, 0.78)	0.73
	PRA+POL vs. PRA+POS	0.00 (-0.69, 0.74)	0.20 (-0.93, 1.34)	0.08 (-0.58, 0.68)	0.79

Figure S1. Publication bias of studies regarding SSI.

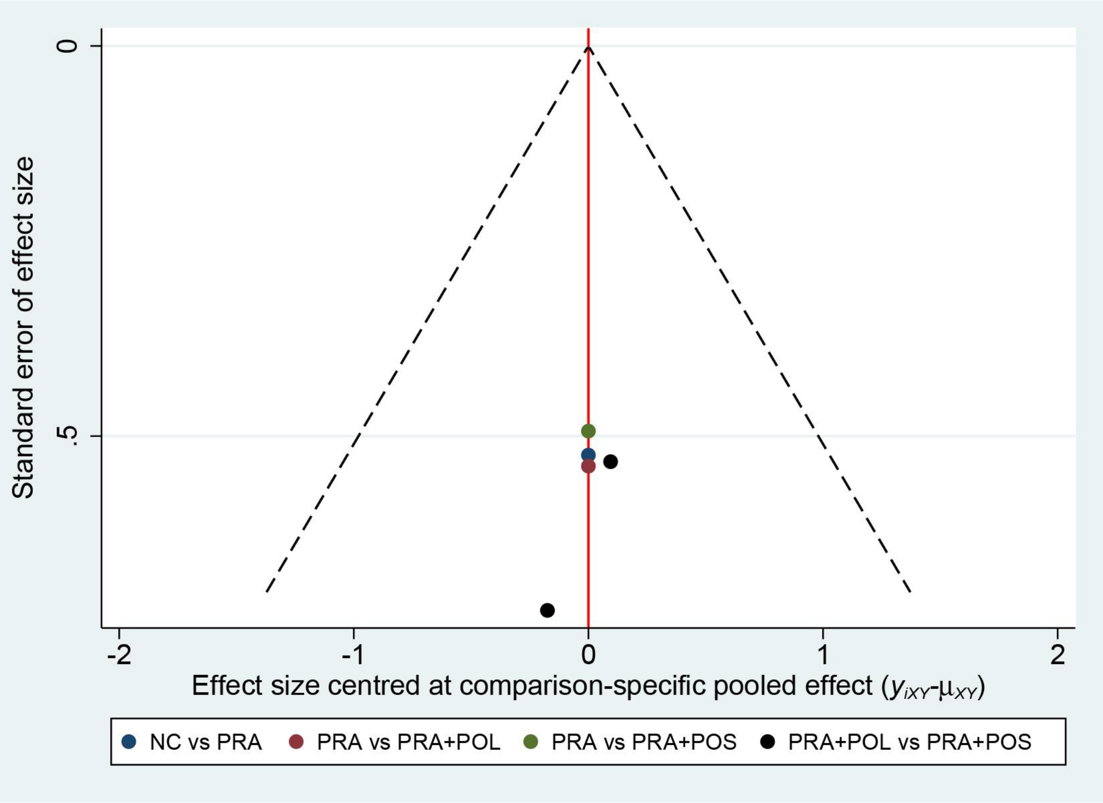

Figure S2. Publication bias testing by funnel plot regarding RSI.

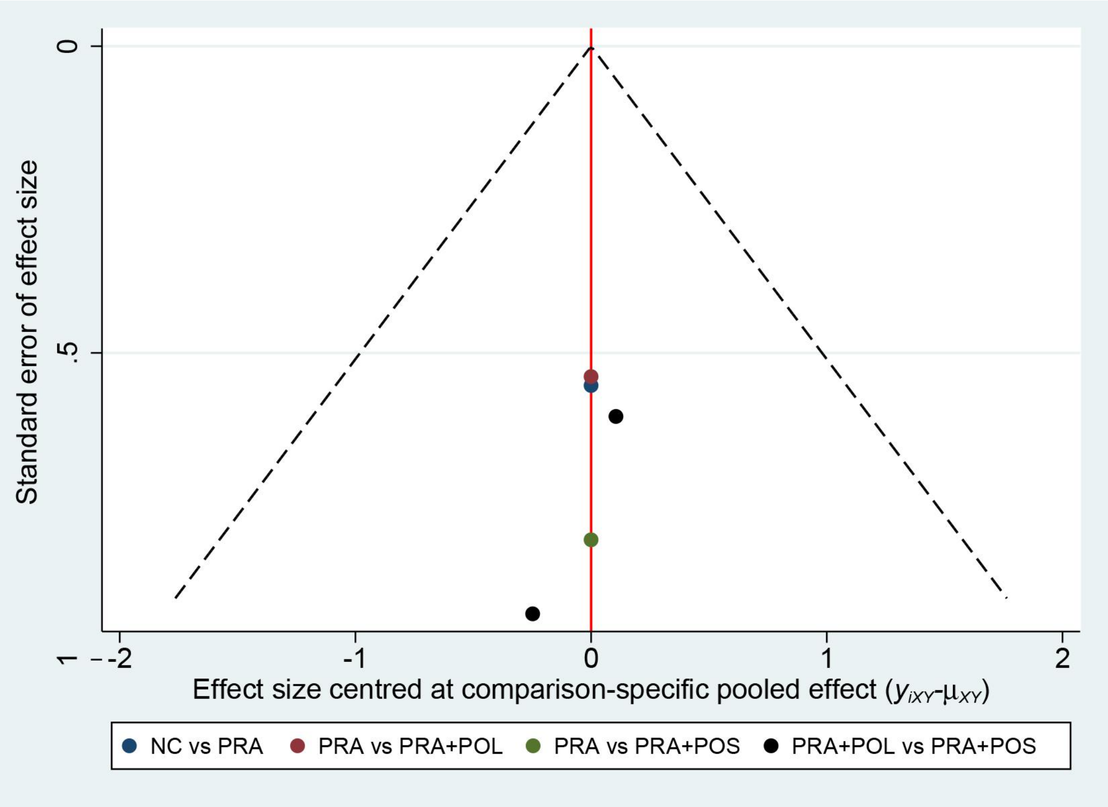

Figure S3. Funnel plot of publication bias examination regarding TI.

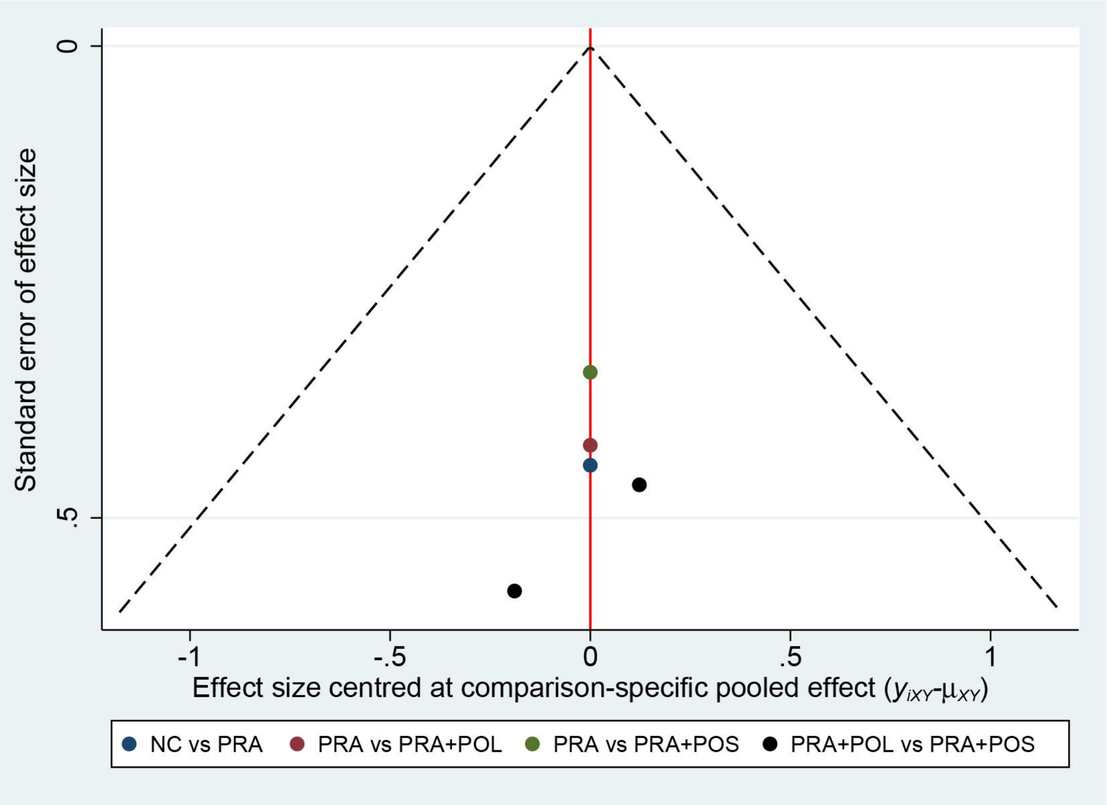

Supplement: Supplemental Digital Content [file medi-98-e16241-s001.pdf]
